# Changes in working hours and burnout levels among physicians: a cohort study

**DOI:** 10.1186/s12960-026-01064-0

**Published:** 2026-04-02

**Authors:** Franziska Ulrike Jung, Erik Bodendieck, Alexander Pabst, Melanie Luppa, Steffi G. Riedel-Heller

**Affiliations:** 1 Institute of Social Medicine, Occupational Health and Public Health (ISAP), Medical Faculty, Leipzig University, Ph.-Rosenthal-Str. 55, 04103 Leipzig, Germany; 2 General Practice, Dresdner Straße 34a, 04808 Wurzen, Germany

**Keywords:** Physicians, Working time, Part-time, Full-time, Burnout, Health care

## Abstract

**Background:**

Currently, the health care system in Germany is facing a serious physician shortage. Previous research reported changes in working hours and a tendency towards a reduction in clinical hours. The aim was to investigate the relationship between changes in working hours and symptoms of burnout in German physicians over two time points.

**Method:**

In 2020, a random sample of physicians from the Federal State of Saxony (Germany) was drawn. The current analyses are based on data from a longitudinal survey conducted in 2020 and 2024. Overall, a sample of *n* = 333 physicians working in both inpatient and outpatient care was investigated using descriptive and regression analyses. In a multivariate analysis predicting burnout symptoms (overall, patient-related, work-related, personal burnout) in 2024, sociodemographic factors such as age, sex and work-related aspects (i.e. medical setting and working hour characteristics), as well as burnout level in 2020, were controlled for.

**Results:**

Overall, 19.2% (*n* = 64) of the sample reported no changes in working hours over 4 years, whereas 27% (*n* = 90) reported an increase in working hours and 53.8% (*n* = 179) reported a decrease in working hours. In fact, a working hour reduction was significantly linked to lower overall burnout, lower personal burnout and lower work-related burnout at follow-up (*p* < 0.001 for all regression models), while controlling for covariates. No significant association between change in working hours and the third burnout dimension—patient-related burnout at follow-up—was found.

**Discussion:**

The present findings emphasize that physician burnout remains a critical issue, especially in the context of clinical working hours. Reducing actual working hours and decreasing overwork may help to improve physician well-being. Future efforts should also focus on optimizing working conditions beyond hours alone—such as increasing schedule flexibility and addressing systemic stressors—to sustainably protect physicians’ health and ensure high-quality patient care.

## Introduction

Worldwide, health care systems are currently facing several challenges, such as financial pressure, an ageing society with increasing health care demands, as well as a shortage of health care professionals [1, 2]. As a result, many professionals, such as physicians, are grappling with symptoms of depression and anxiety, stress-related health issues and burnout throughout their careers [3–6]. Especially physician burnout has been related to poor quality of care and increasing costs for society and health care in terms of turnover intentions and early retirement, as well as effects on patient-related outcomes [7–10]. Although burnout is seen as a complex problem involving many facets, previous research indicates that the high frequency of physicians’ working hours may play a particular role [11, 12]. In Germany, a recent study reported an average working time for physicians of 46 to 49 h per week [13]. Studies from other countries report very similar results, ranging on average between 48 and 51 h per week among physicians [12, 14].

In general, health care professionals are often exposed to irregular schedules, extended working hours and shift work. Several working time characteristics (i.e. overwork hours or less autonomy with regard to working hours) may have a negative impact on physician health and well-being [15, 16]. So far, several studies have shown a decline in cognitive ability when working long hours due to alterations in brain structures, such as higher brain volumes [17, 18]. In addition, working long hours is known to cause mental health problems, such as depression and anxiety [19–21]. Moreover, occupational stress, burnout and sleep problems have also been related to overwork or long work hours [7, 22]. In this context, working hour reduction has become increasingly popular among physicians with different medical backgrounds [12, 23, 24]. Reasons for reducing clinical working time may be related to administrative work, family responsibilities or activities, such as research or teaching [13]. According to a recent review including studies with employers from different sectors, working hour reduction may be associated with improvements regarding physical health, reduced stress and fatigue, better sleep quality and satisfaction with work–life quality [25]. Studies focussing on physicians are underrepresented, but indicate that a decrease in working hours may be associated with lower emotional exhaustion [26], reduced fatigue and higher quality of life, according to a recent review [27].

To date, most evidence on the benefits of reducing working hours among physicians stems from studies focusing on patient-related outcomes, such as patient safety or quality of care [23, 28–30]. In addition, previous intervention studies focused only on the effects of working hour reduction and were limited to a certain number of working hours, with no variation. Longitudinal data can close this gap by helping to understand whether changes in working time are associated with subsequent improvements or deteriorations in physicians’ well-being. In addition, previous studies often focus on contractual working hours. However, excessive working hours are very common in the medical profession. A substantial body of research has examined the adverse effects of long or excessive working hours or shift work on physicians’ health, well-being, and burnout [31, 32]. In contrast, comparatively little is known about how *changes* in working hours—particularly reductions in working time—are associated with physicians’ health outcomes, such as burnout. Therefore, the aim of the current study was to investigate the association between actual working hours and burnout by controlling for several sociodemographic characteristics as well as factors related to the work-setting or working time. To our knowledge, this is the first study that aimed to explore the relationship between burnout symptoms and changes in working time among physicians with different medical backgrounds. Based on data from two time points (2020 and 2024), we hypothesized that a reduction in clinical working hours may be associated with a decrease in burnout symptoms, while an increase in working hours may be associated with a worsening of burnout symptoms over time.

## Method

### Recruitment and study population

The present analysis is part of a longitudinal study exploratorily investigating working hours and retirement patterns among physicians employed in inpatient and outpatient care in the Federal State of Saxony, Germany. At the outset of the project in February 2020, *n* = 2997 physicians were randomly selected from a representative, age-stratified sample of active Saxon physicians with different medical backgrounds and invited to participate via a paper-based questionnaire. The sampling strategy was guided by established methodological principles and involved the construction of nine age-defined cohorts (26–30 years, 31–35 years, and subsequent 5-year intervals). For each cohort, a target of 100 respondents was set to ensure sufficient statistical power using the software G*Power and to allow for an adequate number of participants in the follow-up assessment conducted in 2024. Assuming an expected response rate of approximately 30%, 333 physicians were randomly selected and invited to participate in each age group, resulting in a total of 2997 physicians contacted across all cohorts in 2020. The follow-up survey was conducted in February 2024. A comprehensive description of the overall research project is available elsewhere [33]. The underlying sampling procedure has also been described in greater detail before [34]. At both time points, approximately one-third of physicians contacted by mail replied, resulting in *n* = 987 cases in 2020 and *n* = 979 cases in 2024. Only physicians who participated in both baseline (2020) and follow-up (2024) surveys were included in the current analyses (*n* = 372, [35]). Statistically significant differences were observed between the full sample and the sub-sample in terms of sex and age (*p* < 0.001 for both variables), with the sub-sample comprising a higher proportion of younger and female physicians. Exclusion criteria included being older than 67 years at baseline (i.e. statutory retirement age for physicians in Germany, *n* = 20), unemployment or maternal leave at baseline or follow-up (*n* = 9) or implausible or missing data regarding working hours (*n* = 10). Consequently, the final analytical sample consisted of *n* = 333 physicians. The study was approved by the Ethics Committee of the Medical Faculty at Leipzig University (Reference No. 478/19-ek) prior to data collection.

### Instruments

In addition to sociodemographic variables such as age, sex (dichotomous: female or male), marital status, and parenthood (yes/no), the questionnaire included several items to assess the participant’s current working conditions. In this context, participants were asked whether they work in a hospital (answer format: yes/no), whether they work in shifts (answer format: yes/no), whether they work on weekends and whether they are self-employed or employed (answer categories: employed—fixed-term, employed—permanent, self-employed).

To capture working time hours (actual working hours, including overwork and on-call service), participants were asked how many hours they were currently working per week. Based on their answers at both time points (2020 and 2024), the difference between working hours was calculated (hours_2020_—hours_2024_) for each participant (in hrs/week) in order to derive possible changes with regard to weekly working hours.

Burnout was assessed using the Copenhagen Burnout Inventory (CBI) [36], a validated instrument designed to measure perceived stress related to professional activity. The CBI comprises 19 items rated on a five-point Likert scale and has been specifically adapted and used for professionals in the health care sector (i.e. aligned to the work with patients instead of clients) [37]. Example items cover information with regard to the following three subscales: personal burnout (e.g. “How often do you feel tired?”; response range: all the time to never/almost never), work-related burnout (e.g. “Does your work emotionally exhaust you?”; response range: very strongly to not at all), and patient-related burnout (e.g. “Does working with patients emotionally exhaust you?”; response range: very strongly to not at all). An overall burnout score was calculated as the sum of all items from the personal, work-related, and patient-related burnout subscales of the CBI, with higher scores indicating greater overall burnout severity [36, 38]. In the current study, Cronbach alpha reliability coefficients of the CBI and its subscales at both baseline and follow-up were high (range: 0.86–0.94).

### Statistical analysis

Overall, data were first analysed descriptively with regard to sociodemographic and work-related characteristics at baseline, as well as working hours and burnout symptoms at baseline and follow-up. Means with standard deviations or frequencies with percentages are shown, as appropriate. Differences in burnout scales between baseline and follow-up were tested using Wilcoxon matched-pairs signed-rank tests.

Secondly, four separate negative binomial multivariate regression models were applied with burnout symptoms at follow-up as the dependent variable (Model 1: overall burnout; Model 2: personal burnout; Model 3: work-related burnout; Model 4: patient-related burnout, continuous variables). Burnout scores were treated as continuous outcomes and modelled using negative binomial regression due to their skewed distribution and evidence of overdispersion. Change in working time hours (continuous) was included as the main predictor. In line with the multidimensional conceptualization of burnout underlying the CBI, all three subscales (personal, work-related, and patient-related burnout) as well as an overall burnout score were examined, as changes in working hours may affect both work-specific and general exhaustion or fatigue. All four models were adjusted for the following covariates: age, sex, parenthood, marital status, working in a hospital, shift work, weekend work, and working status. Marital status was dichotomized (0 = single/divorced/widowed/married but living apart and 1 = married/living with a partner). Each model additionally included the corresponding baseline burnout score as a control variable (e.g. personal burnout at baseline was included in the model predicting personal burnout at follow-up). Multicollinearity was assessed using variance inflation factors (VIFs). Results of the negative binomial regressions are reported as incidence-rate ratios (IRR), with p-values and 95% confidence intervals. A p-value less than 0.05 was considered statistically significant. All statistical analyses were conducted using Stata SE Version 16 [39].

## Results

Baseline characteristics with regard to sociodemographic and work-related information of the sample are shown in Table 1. The mean age was 45 years (SD 10.8); 65% were female; 59% were working in inpatient care; and half of the participants (49.9%) were permanently employed at baseline.

**Table 1:** Sociodemographic and work-related characteristics of the sample at baseline and at follow-up (*n* = 333)

Variable	Baseline
Age	Mean: 44.3 (SD: 10.8) median: 44.0 range: 25–66
Sex	*n* (%)
Women	216 (64.9%)
Men	117(35.1%)
Care for children	*n* (%)
Yes	255 (76.6%)
No	76 (22.8%)
Missing	2 (0.6%)
Marital status	*n* (%)
Single	32 (9.6%)
Living with a partner	67 (20.1%)
Married	210 (63.1%)
Married, but living apart	7 (2.1%)
Widowed	3 (0.9%)
Divorced	14 (4.2%)
Working in a hospital	*n* (%)
Yes	195 (58.6%)
No	138 (41.4%)
Shift work	*n* (%)
Yes	53 (15.9%)
No	274 (82.3%)
Missing	6 (1.8%)
Working on weekends^1^	*n* (%)
Yes	244 (73.3%)
No	78 (23.4%)
Missing	11 (3.3%)
Working contract	*n* (%)
Employed (fixed-term)	81 (24.3%)
Employed (permanent)	166 (49.9%)
Self-employed	85 (25.5%)
Missing	1 (0.3%)

*SD* standard deviation;

^1^ “yes” implies that physicians work on the weekend for at least once per month, “no” implies that physicians never work on the weekend

Overall, the actual working time—including overwork and on-call service—ranged from 14 to 92 h/week at baseline (mean: 49.0; SD: 12.4) and from 7 to 97 h/week at follow-up (mean: 45.5, SD: 12.9), indicating a general working hour reduction between 2020 and 2024.

Overall, 19.2% (*n* = 64) of the sample reported no changes among working hours, whereas 27% (n = 90) reported an increase in working hours (mean change: 7 h/week), and 53.8% (*n* = 179) reported a decrease in working hours (mean change: 10 h/week) between baseline and follow-up.

Table 2 summarizes the change among burnout (overall and regarding the three subscales). No significant differences were found regarding baseline and follow-up scores.

**Table 2:** Comparison of burnout sum scores (overall and subdimensions) between baseline (2020) and follow-up (2024)

Variable	Baseline mean (SD)	Follow-up mean (SD)	*p*-value^1^
Burnout—overall score	33.8 (SD: 15.6) range: 5.3–90.8	34.6 (SD: 17.0) range: 4.0–86.8	*p* = 0.386
Personal burnout	44.0 (SD: 17.7) range: 4.2–100	45.6 (SD: 19.8) range: 0–100	*p* = 0.120
Work-related burnout	34.2 (SD: 16.9) range: 3.6–92.9	34.8 (SD: 19.0) range: 0–92.9	*p* = 0.600
Patient-related burnout	23.5 (SD: 19.5) range: 0–95.8	24.1 (SD: 19.3) range: 0–100	*p* = 0.457

*SD* standard deviation;

^1^Wilcoxon signed-rank test; *n* = 333

Results of the multivariate regression models with overall burnout at follow-up as the dependent variable are shown in Table 3 (Model 1). According to the results, a change in working hours is negatively, but significantly related to burnout at follow-up (*p* < 0.001). In other words, when adjusting for covariates, a reduction in weekly working hours between baseline and follow-up (as indicated by an increase in ∆ working time) was associated with a 1% lower overall burnout score among physicians at follow-up. In addition, burnout at baseline (*p* < 0.001) and age (*p* = 0.009) were significantly associated with burnout at follow-up.

**Table 3:** Negative binominal regression with overall burnout as the dependent variable (Model 1)

	Model 1: burnout (overall)	
	IRR	*p* (95% CI)
∆ working time^1^	**0.990**	**< 0.001 (.985; .994)**
Overall burnout (baseline)	**1.020**	**< 0.001 (1.017; 1.024)**
Age	**0.992**	**0.009 (.987; .998)**
Sex (ref. female)	1.019	0.683 (.931; 1.115)
Having children (ref. yes)	0.932	0.261 (.825; 1.053)
Marital status^2^	0.985	0.816 (.869; 1.117)
Working in a hospital (ref. yes)	1.103	0.132 (.971; 1.253)
Shift work (ref. no)	1.087	0.187 (.960; 1.230)
Working on weekends (ref. no)	0.987	0.832 (.878; 1.110)
Working status (ref. permanent)	*χ*^2^ (2) = 1.21, *p* = 0.547	
Fixed-term	0.933	0.293 (.820; 1.062)
self-employed	1.021	0.764 (.892; 1.168)

Bold values indicates the statistical significance, e.g. *p*-value < 0.001

*IRR* incidence rate ratio, *CI* confidence interval

^1^Continuous variable, indicating the difference of actual working hour/week between baseline and follow-up, the greater the score, the greater the working hour reduction

^2^Reference: single/divorced/widowed/married but living apart; the model (*n* = 291) was controlled for baseline covariates

In addition, each of the three subdimensions of burnout was analysed separately (see Tab. 4).

**Table 4:** Negative binominal regression models with each burnout dimension as the dependent variable

	Model 2: personal burnout		Model 3: work-related burnout		Model 4: patient-related burnout	
	IRR	p (95% CI)	IRR	p (95% CI)	IRR	p (95% CI)
∆ working time^1^	**0.992**	**< 0.001 (.988; .996)**	**0.989**	**< 0.001 (.984; .994)**	.992	0.087 (.982; 1.001)
Burnout dimension (baseline)^2^	**1.015**	**< 0.001 (1.013; 1.018)**	**1.020**	**< 0.001 (1.016; 1.023)**	**1.028**	**< 0.001 (1.023; 1.034)**
Age	0.995	0.098 (.989; 1.001)	**0.992**	**0.014 (.985; .998)**	.996	0.534 (.985; 1.008)
Sex (ref. female)	0.977	0.591 (.895; 1.065)	1.040	0.469 (.936; 1.155)	1.190	0.078 (.981; 1.444)
Having children (ref. yes)	0.969	0.0.600 (.859; 1.092)	0.939	0.393 (.813; 1.085)	0.941	0.648 (.724; 1.223)
Marital status^3^	0.964	0.549 (.857; 1.086)	0.930	0.325 (.804; 1.075)	0.965	0.796 (.737; 1.264)
Working in a hospital (ref. yes)	1.108	0.094 (.983; 1.250)	1.113	0.155 (.960; 1.291)	1.088	0.547 (.828; 1.429)
Shift work (ref. no)	1.008	0.895 (.895; 1.136)	**1.161**	**0.045 (1.003; 1.343)**	1.130	0.365 (.867; 1.472)
Working on weekends (ref. no)	1.022	0.694 (.917; 1.140)	1.008	0.913 (.881; 1.52)	0.863	0.239 (.675; 1.103)
Working status (ref. permanent)	χ^2^ (2) = 0.20, p = 0.904		χ^2^ (2) = 3.15, p = 0.207		χ^2^ (2) = 0.95, p = 0.623	
Fixed-term	1.018	0.785 (.896; 1.156)	0.872	0.077 (.749; 1.015)	1.004	0.980 (.764; 1.318)
self-employed	1.024	0.723 (.900; 1.164)	0.987	0.873 (.840; 1.159)	1.151	0.332 (.867; 1.528)

Bold values indicates the statistical significance, e.g. *p*-value < 0.001

*IRR* incidence rate ratio, *CI* confidence interval; all models were controlled for baseline covariates

^1^Continuous variable, indicating the difference of actual working hour/week between baseline and follow-up, the greater the score, the greater the working hour reduction

^2^Burnout dimension, specifically used in each model: Model 2: personal burnout at baseline (*n* = 309), Model 3: work-related burnout at baseline (*n* = 307); Model 4: patient-related burnout at baseline (*n* = 303); ^3^ = reference: single/divorced/widowed/married but living apart

According to the results of Model 2, a working hour decrease was significantly associated with a decrease in personal burnout at follow-up (*p* < 0.001). In addition, greater personal burnout at baseline was related to greater personal burnout at follow-up (*p* < 0.001). Similar results were obtained for Model 3, hence work-related burnout. Here, a working hour reduction was significantly associated with less work-related burnout at follow-up (*p* < 0.001). In addition, higher age (*p* = 0.014) was related to higher work-related burnout at follow-up, whereas greater burnout at baseline (*p* < 0.001) and working in shifts (*p* = 0.045) were linked to greater work-related burnout at follow-up. Interestingly, Model 4 did not reveal a significant association between working hour change and patient-related burnout at follow-up (*p* = 0.087). Only higher patient-related burnout at baseline (*p* < 0.001) was related to higher patient-related burnout at follow-up (see Tab. 4).

## Discussion

The aim of the current study was to explore the impact of working time change in physicians on the severity of burnout symptoms. In our sample, more than half of physicians reported a reduction in working hours between 2020 and 2024, consistent with previous studies documenting declining clinical workloads across medical specialties [8, 12, 40]. From an individual-level perspective, reduced working hours may be beneficial, as our findings suggest that decreases in working time are associated with lower burnout symptoms. At the system level, widespread reductions in working hours may contribute to workforce capacity challenges, particularly in the context of rising health care demands driven by population ageing and increasing prevalence of chronic disease [41–43]. Importantly, addressing these challenges by increasing individual physicians’ working hours may be neither feasible nor desirable. Instead, strategies such as workforce expansion, task redistribution, and organizational interventions to prevent burnout may be more sustainable approaches.

Previous studies have shown that demanding work schedules (e.g. overlong hours, shift work, working on weekends) can negatively affect the health and well-being of physicians. Focusing on burnout is important, because physician burnout has been related to early retirement and turnover [44] and generally increases costs for health care and society [45, 46]. In this context, research has shown that burnout (measured by emotional exhaustion) was associated with a higher likelihood of working time reduction [8]. Building up on these previous findings, the current study results indicate that a decrease in clinical working hours was accompanied by less overall burnout, as well as less personal and work-related burnout at follow-up, when controlling for baseline burnout scores and other relevant covariates. This is underlined by evidence from longitudinal and cross-sectional studies that investigated the link between working hours and burnout symptoms or burnout risk [47], although working time may only be one of many job stressors among physicians [48]. On the one hand, reducing working hours may increase recovery and sleep opportunities, while at the same time reducing exhaustion and balancing physiological processes [31, 47, 49, 50]. This has also been confirmed by a recent 4-day-workweek-intervention, where work time reduction was correlated with reduced sleep problems and decreased fatigue [51]. In addition, work–life conflict has been identified as a major contributor to physician burnout [8, 52, 53]. Reducing working hours may positively contribute to work–life balance and therefore reduce overall burnout over time. A third explanation may be related to the effort-reward-imbalance model [54]. Previous research has shown that a higher frequency of clinical working hours per week is related to higher effort-reward imbalance as well as greater burnout [55]. Here, a reduction of working hours over ten years was accompanied by less effort–reward imbalance and a trend towards a reduction in burnout risk. However, this study included physicians working in intensive care only and two independent cross-sectional surveys. Therefore, no comparison over time was possible.

Beyond working hours alone, burnout risk is likely shaped by the interaction between time demands and other work-related stressors. Long working hours may be particularly harmful in contexts characterized by high administrative burden, frequent interruptions, emotional strain, or limited job control. Conversely, extended working hours may be more tolerable when accompanied by adequate resources, autonomy, and supportive organizational structures. The current findings suggest that changes in working hours may reflect physicians’ attempts to cope with cumulative job demands rather than the effect of working time in isolation. Future studies should therefore examine interaction effects between working hours and specific job demands to better identify high-risk constellations and inform targeted organizational interventions.

According to our study, no significant relationship could be found between working hour change and patient-related burnout. This is an interesting finding and supports the assumption that it is not the amount of time that physicians spend within clinical settings, but rather the strain and fatigue that may occur when dealing with patients. In other words, burnout may not always translate to patient interaction deficits. Some studies found that even highly burned-out physicians maintain patient care standards—possibly driven by professional norms or conservation of patient responsibility even at personal cost [56, 57]. Patient-related burnout (i.e. exhaustion due to direct patient care) may be more affected by qualitative stressors—empathic strain, emotional labour and moral distress—than by volume of hours. In addition, there is evidence that certain job stressors, such as work hours or inadequate work-free time may be linked to personal and work-related burnout domains, but no association with patient-related burnout has been demonstrated among physicians so far [48, 58], which is consistent with the current findings.

In addition, several other factors have been shown to be related to burnout within the study context. Higher age was related to lower burnout scores (overall and work-related burnout). There are several studies reporting a negative effect of age on burnout [59–61]. This could be explained by greater work experience, more autonomy with regard to decision-making due to higher positions and possible selection effects, as those being more vulnerable to burnout may leave patient care earlier. Another factor, that proved significant in the regression analysis, was shift work, indicating that physicians who work in shifts reported higher work-related burnout at follow-up. This has been demonstrated by similar studies within health care settings before [49, 62, 63] and may be explained by two reasons. First of all, shift work often includes night shifts or long hours, which may increase workload and stress and lead to decreased recovery time [31, 64]. On the other hand, working in shifts may interfere with family or private life, limiting the protective factors against burnout [53, 65].

In general, offering more autonomy and flexibility in terms of working hour schedules may not only help to reduce burnout and increase satisfaction [16, 32], it may also be a possibility to attract a larger medical workforce (i.e. female physicians and physicians that have already reached retirement age). As a result, the physician shortage might be diminished, counteracting the increasing demands of health care. Organizational interventions, such as job crafting may also be relevant in this context, as it emphasizes the active adaptation of work tasks to personal needs and resources [66]. Physicians who are able to align their reduced working hours with a corresponding adjustment in workload and task design may not be so vulnerable to developing persistent burnout [66, 67]. Since physicians with reduced working time often have to balance clinical and nonclinical obligations, it is imperative to consider the necessity of enhanced institutional support to ensure the sustained clinical effectiveness of these medical professionals. Otherwise, patients’ safety may be threatened and physicians may suffer from high workload and distress. In addition, it has been shown that working less than full-time may be related to worse patient outcomes in terms of 30-day mortality compared to full-time work [68]. Besides, it has been shown that continuity of care, which may be important in terms of therapeutic outcomes and compliance, may be negatively affected if physicians decide to work fewer clinical hours and are therefore not available for appointments on a regular basis [28, 69].

## Limitation

To our knowledge, this is the first study investigating the relationship between burnout and change in working hours among physicians by comparing data of two different time points. Our study’s design (comparing change over time) is particularly valuable, as it isolates the association between *change* in working hours and *later* burnout outcomes—this adds weight to the temporal relationship, reducing reverse‐causation concerns. However, the study does have some limitations as well. For instance, future studies may want to include more than two measurement time points or real-time tracking in order to investigate how burnout symptoms change in relation to changes in clinical working hours (e.g. continuously decreasing or changing between increase and decrease). There may be other short-term effects on burnout followed by working time reduction or increase, that were overlooked by the current study. In addition, the sample is rather small and a differentiation between medical professions was not possible. At both time points under investigation, there may be a selection bias, as some physicians may have been too stressed or burned out to take part in this study. Besides, the study period overlapped with the COVID-19 pandemic and no pre-pandemic data was included for comparison. Pandemic-related changes in clinical demands, staffing, and working time may therefore have contributed to both reductions in working hours and elevated burnout symptoms observed in this study [70, 71]. Prospective research should explore mediating variables such as workload intensity per hour, administrative burden, or organizational support, as these may modulate the relationship between changes in working hours and symptoms of burnout.

## Conclusion

Working hour reductions are very common among physicians in Germany with different medical backgrounds. At the same time, the results suggest that reduced working hours may lead to improvements in terms of burnout or perceived personal as well as work-related stress. However, other aspects of working time—such as flexibility in scheduling—or additional harmful working conditions should be considered when developing strategies to protect physicians from long-term health impairments and turnover. At the same time, reducing hours alone may be insufficient unless organizational factors (e.g. staffing, administrative load, effort–reward balance) are addressed.

## Data Availability

The data that support the findings of this study are available from the corresponding author [F.U.J.], upon reasonable request due to ethical and privacy restrictions.
